# A Closed Loop Brain-machine Interface for Epilepsy Control Using Dorsal Column Electrical Stimulation

**DOI:** 10.1038/srep32814

**Published:** 2016-09-08

**Authors:** Miguel Pais-Vieira, Amol P. Yadav, Derek Moreira, David Guggenmos, Amílcar Santos, Mikhail Lebedev, Miguel A. L. Nicolelis

**Affiliations:** 1 Department of Neurobiology Duke University, Durham, NC 27710, USA; 2 Centro de Investigação Interdisciplinar em Saúde, Instituto de Ciências da Saúde, Universidade Católica Portuguesa, Porto, Portugal; 3 Instituto de Ciências da Vida e da Saúde, Universidade do Minho, Braga, Portugal; 4 Department of Biomedical Engineering Duke University, Durham, NC 27710, USA; 5 Duke Center for Neuroengineering Duke University, Durham, NC 27710, USA; 6 Department of Psychology and Neuroscience Duke University, Durham, NC 27710, USA; 7 Edmond and Lily Safra International Institute of Neuroscience of Natal, Natal, Brazil.

## Abstract

Although electrical neurostimulation has been proposed as an alternative treatment for drug-resistant cases of epilepsy, current procedures such as deep brain stimulation, vagus, and trigeminal nerve stimulation are effective only in a fraction of the patients. Here we demonstrate a closed loop brain-machine interface that delivers electrical stimulation to the dorsal column (DCS) of the spinal cord to suppress epileptic seizures. Rats were implanted with cortical recording microelectrodes and spinal cord stimulating electrodes, and then injected with pentylenetetrazole to induce seizures. Seizures were detected in real time from cortical local field potentials, after which DCS was applied. This method decreased seizure episode frequency by 44% and seizure duration by 38%. We argue that the therapeutic effect of DCS is related to modulation of cortical theta waves, and propose that this closed-loop interface has the potential to become an effective and semi-invasive treatment for refractory epilepsy and other neurological disorders.

Drug-resistant epilepsy constitutes about 22.1% of the total cases of epileptic patients^1^. Historically, these cases have been treated with surgery^2^, but more recently electrical neurostimulation has emerged as a potential alternative therapeutic approach^3^. Deep brain^4^, vagus^5^, and trigeminal^6^,^7^ nerve stimulation, a procedure pioneered in our laboratory, have been proposed over the past decade as new alternatives to treat refractory epilepsy. However, each of these three alternative therapies has its advantages and disadvantages. For example, deep brain stimulation (DBS) has a success rate of 60% in patients with refractory epilepsy^8^, but requires extremely invasive brain surgery. Therefore, a smaller number of patients will be eligible for DBS when compared to the other alternative therapies^9^. Trigeminal nerve stimulation (TNS) is far less invasive than DBS, but has a success rate of only 30.2%^6^. Lastly, vagus nerve stimulation (VNS) is also less invasive than DBS, but its success rate is the lowest among all three therapies at 24–28% in randomized clinical trials^10^,^11^.

Electrical stimulation of the posterior funiculus, also known as the dorsal column, of the spinal cord is a semi-invasive method^12^ which we have demonstrated to be effective for Parkinson’s disease (PD) treatment in rodents^13^,^14^ and primates^15^, and others have shown to be effective in Parkinsonian patients^16^,^17^. Remarkably, the neurophysiological hallmark of Parkinson’s disease in animal models is defined by hypersynchronized neuronal activity in the beta band of local field potentials (LFPs)^13^,^15^. The LFP patterns observed during these periods of hypersynchronized neuronal activity in Parkinson’s disease resembled some of the patterns of hypersynchronized neuronal activity previously reported in pentylenetetrazol (PTZ) injected rats^18^. This latter similarity and the fact that this neuronal hypersynchronization can be specifically disrupted by DCS^13^,^14^,^15^ led us to hypothesize that DCS could be used as an alternative treatment for chronic refractory epilepsy. Although a recent study has demonstrated that DCS improved seizure related activity in anesthetized rats injected with PTZ^19^, the full clinical potential of DCS can only be truly addressed in awake animals with DCS being applied in a closed loop mode (i.e. triggered only when a seizure is detected by an alternative measurement, such as cortical neuronal recordings). While PTZ injection may not be the best model to represent the subset of patients with refractory epilepsy^20^, it has provided the most promising results of DCS as an alternative to current neurostimulation techniques^19^.

Here we developed a closed-loop brain-machine interface (BMI) that utilized chronic cortical implants to detect seizure activity in awake, freely moving PTZ-treated rats (Fig. 1A,B). This BMI also allowed DCS to be delivered using the method we previously developed to suppress Parkinson’s symptoms in rodents^13^. Overall, we observed that this closed-loop BMI substantially reduced the frequency and duration of seizure episodes.

## Results

**Figure 1:**
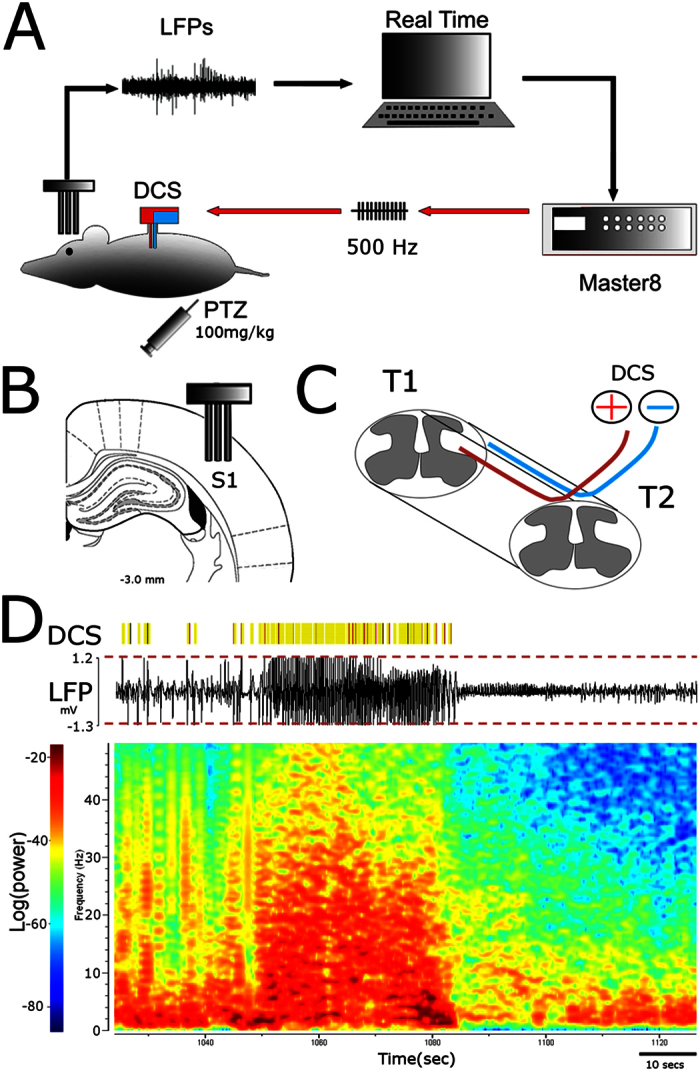
Closed loop brain-machine interface setup. (**A**) Local Field Potentials recorded from primary somatosensory cortex are analyzed in real time. High amplitude signals trigger the microstimulator (Master8) which will deliver an electrical pattern to the dorsal columns (DCS). (**B**) Recording electrodes placement^44^. (**C**) Stimulating electrodes placement (resting in the epidural space between the vertebrae and the spinal cord). (**D**) Raw LFP recording with multiple crossings of pre-established threshold (red dashed lines). The yellow bars indicate DCS delivered whenever the threshold was crossed. Bottom: Spectrogram depicting a seizure episode.

A total of 10 rats (six male and four female) were implanted with stimulation and recording electrodes. Several days after the animals recovered from this implantation surgery, they were injected with PTZ and the efficacy of our closed-loop BMI in suppressing seizure episodes and reducing their duration was examined in 30 experimental sessions. Cortical microelectrode implants were placed in the primary somatosensory cortex (S1) and used for local field potential recordings (LFPs). Dorsal column stimulation electrodes were placed at the level of vertebral T1-T2 segments)^13^,^14^ (Fig. 1C). Two types of experiments were conducted in these 10 animals.

### Experiment 1: BMI-On versus BMI-Off

In the first experiment (6 male and 3 female rats; 23 experimental sessions), seizure parameters were measured in PTZ-treated rats either with or without DCS driven by the closed loop BMI (BMI-On and BMI-Off sessions, respectively). In BMI-On sessions, each time a seizure detection threshold was crossed (Fig. 1D), five trains of 200 electrical biphasic pulses (100–200 uAmp) were delivered at the frequency of 500 Hz to the dorsal column. In BMI-Off sessions, the recording and stimulation equipment were connected the same way, but no DCS was delivered to the animals.

Injection of PTZ induced characteristic spike and wave discharges (SWDs)^21^ that were very evident in cortical LFP recordings (Fig. 1D) and triggered body twitches as their main behavioral manifestation. SWD frequency typically increased until a seizure episode occurred (Fig. 1D). Once a seizure was detected, our BMI delivered the DCS after each SWD with a 50 ms delay.

**Figure 2:**
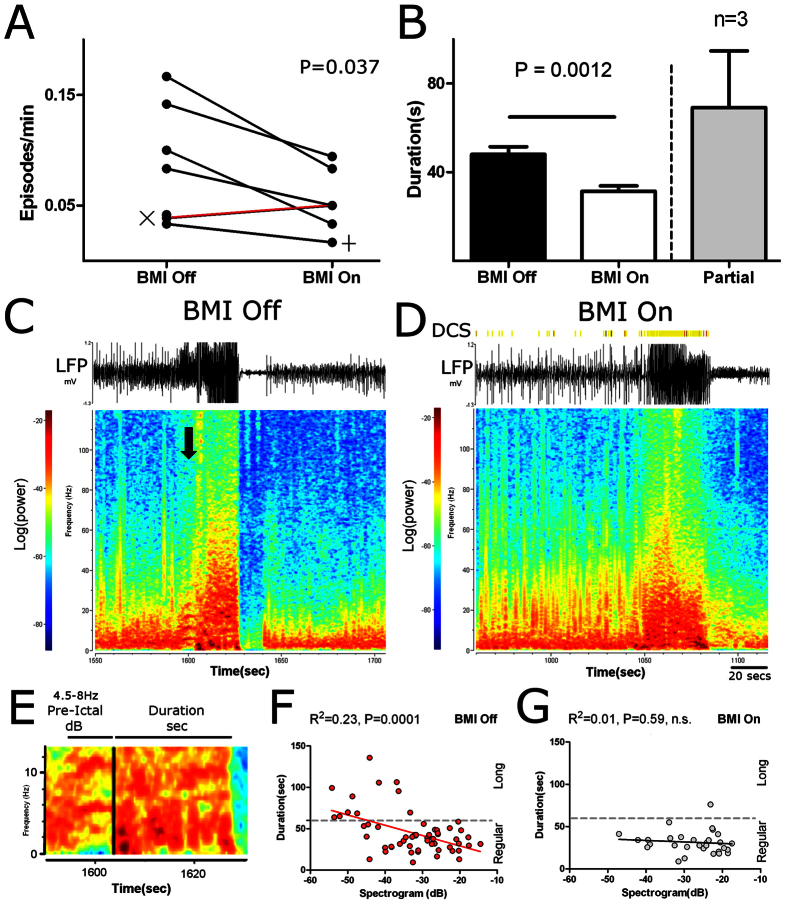
DCS improves seizure related activity. (**A**) DCS reduced the frequency of seizure episodes. The only case where the frequency of seizures was not reduced (red line, Fig. 2A), corresponds to a session that ended earlier due to technical problems. Symbols X and + correspond each one to a rat with a single BMI-Off or -On session. (**B**) DCS reduced seizure duration. ‘Partial’ indicates seizures where the BMI was activated only during a fraction of the episode. (**C**,**D**) Examples of raw LFP signals and corresponding spectrogram for a BMI-Off and a BMI-On session. During BMI-Off sessions, pre-ictal activity (approximately 1600 seconds) presented a characteristic signature pattern (see text for details). (**E**) Detail of BMI-Off session presented in C (color code as above). (**F**,**G**) In BMI-Off sessions, the pre-ictal theta frequency signal was a good predictor of seizure duration, however during BMI-On sessions, DCS specifically disrupted this signal. Also, note that long seizures (≥60 secs) were mostly absent during BMI-On sessions.

Comparison of the BMI-On and BMI-Off sessions showed that closed loop DCS affected multiple physiological parameters (Fig. 2A–G). In particular, DCS reduced the overall number of seizure episodes by 44% (BMI-On: 0.05 ± 0.01 episodes/min; BMI-Off: 0.09 ± 0.02 episodes/min; Paired Samples test t = 2.816, df = 5; P = 0.0373; Fig. 2A) as well as the number of SWDs by 72% (BMI-On: 1.8 ± 0.3 SWD/min; Off: 6.5 ± 2.6 SWD/min; Wilcoxon signed-rank test = 21; P = 0.0313). Additionally, DCS reduced seizure duration by 34.86% (BMI-On: 31.39 ± 2.4 secs; Off: 48.19 ± 3.5 secs; Min: 9 secs; Max: 136 secs; Mann-Whitney U = 551.5; P = 0.0012, Fig. 2B; Partial indicates episodes where the BMI failed to deliver DCS). No differences were found in seizure episode characteristics when rats, tested in the same conditions, were compared across consecutive sessions (paired samples t-test; Duration: T = 0.3314, df = 4; P = 0.7570, n.s.; Frequency: T = 0.48, df = 4; P = 0.67, n.s,), suggesting that the differences between BMI-On and –Off sessions were not due to repeated PTZ administration.

Further analysis of the distribution of seizure episode durations showed that DCS negatively skewed this distribution, meaning that long seizure episodes (longer than 60 s) became much less frequent (BMI-On: 1/30 = 3.3% episodes; BMI-Off: 11/62 = 17.74% episodes; Fisher’s exact test: P = 0.048; compare Y axis values in Fig. 2 between F,G).

Frequency spectral analysis indicated that DCS specifically disrupted the LFP spectral pattern that preceded the onset of each seizure episode^22^ in PTZ treated rats. This LFP pattern consisted of an elevated theta band (~4 Hz to 8–10 Hz), which often appeared as a parabola^22^. These PTZ-related theta episodes, which usually lasted approximately 5–10 s (compare Fig. 2 panels C,D), occurred in a very narrow range of frequencies and occasionally appeared in higher harmonic frequencies (see arrow in Fig. 2Calso 2E). Thus, although pre-ictal activity very often included other bands, spectrogram changes associated with the period occurring immediately before the seizure episode most reliably appeared in the theta frequency. During BMI-On sessions, this PTZ-induced elevated theta band pattern was disrupted. This means that, after the delivery of DCS, the specific parabola pattern was no longer present even when this frequency band still presented a high potency signal. The main effect observed was an increase of LFP power in a wide theta range (4.5–8 Hz) (BMI-On: −27.57 ± 1.4 dB; BMI-Off: −33.01 ± 1.3 dB; t = 2.64, df = 90; P = 0.0098; also see right shift in X axis values in Fig. 2F,G). Lastly, DCS also induced longer periods with reduced pre-ictal theta band power (BMI-On: 2.81 ± 1.81 secs; BMI-Off: 1.51 ± 0.13 secs; Mann-Whitney U = 306; P = 0.0038). Thus, DCS induced a reduction in the proportion of long seizure episodes, an increase in theta power and range (compare Long and Regular in Fig. 2F,G), and allowed for longer periods with low power in the theta band.

These findings suggest to us that the elimination of the theta pattern by DCS may have accounted for the mechanism that led to seizure reduction. In support of this theory, we observed that theta band power during the pre-ictal period was a good predictor of longer seizure duration in BMI-Off sessions (F_1,55_ = 17.09; R^2^ = 0.23;P < 0.0001: see Fig. 2E,F). By contrast, during BMI-On sessions, theta band power was no longer correlated to seizure duration (BMI-On: F_1,28_ = 0.32; R^2^ = 0.01;P = 0.579, n.s.; Fig. 2G).

### Experiment 2: Mixed BMI on and off episodes within a session

**Figure 3:**
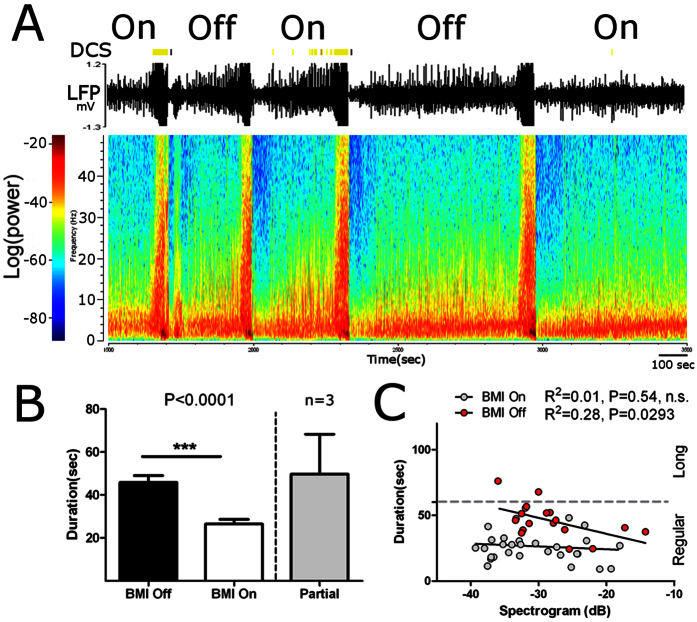
Intermittent delivery of DCS improves seizure related activity. (**A**) Example of session where the BMI was turned On or Off in successive seizure episodes. (**B**) DCS reduced seizure duration. Partial indicates seizure episodes where DCS was delivered only in a fraction of the episode. (**C**) In BMI-Off episodes, the pre-ictal theta frequency signal was a good predictor of seizure duration. During BMI-On episodes, DCS specifically disrupted this signal. Also, note that long seizures (≥60 secs) were absent during BMI-On episodes and during BMI-Off episodes as well (see text for details).

To test how fast our BMI became effective in reducing PTZ induced seizures, we turned the BMI on and off periodically within the same experimental session. We called these experiments the mixed sessions (N = 7 rats, 4 male and 3 female in seven sessions; see Fig. 3A). Seizure episode durations now varied between 9 and 76 seconds. Under these conditions, we found that our closed-loop BMI still drastically reduced episode duration by 42.15% (BMI-On: 26.5 ± 2.1 secs; BMI-Off: 45.81 ± 3.2 secs; Mann-Whitney U = 74; P < 0.0001; see Fig. 3B; Partial indicates episodes where the BMI failed to deliver DCS).

As in the case of the first experiment, pre-ictal theta band power was a good predictor of seizure duration in the mixed sessions when the BMI was off (F_1,15_ = 5.80; R^2^ = 0.28; P = 0.0293; Fig. 3C). Once again, when the BMI was on, the theta band power no longer correlated with seizure duration (BMI-On: F_1,28_ = 0.38; R^2^ = 0.01; P = 0.54, n.s.). Conspicuously, analysis of long seizure episodes (i.e. ≥60 seconds) now revealed that turning the BMI on in a fraction of the seizure episodes significantly reduced the number of these long seizures even when the BMI was off (BMI-On: 0/30 = 0% episodes; BMI-Off: 2/17 = 11.8%; Fisher’s exact test: P = 0.145, n.s.; also see Fig. 3C, compare Regular to Long). This finding suggested that, during the course of a PTZ session, the delivery of the DCS pattern during one seizure episode could, to some extent, affect the characteristics of the following episode^13^,^14^,^15^, even if no DCS was delivered at that particular episode. In other words, we found evidence for a long-lasting effect of DCS, similar to what we had reported before when we used DCS to treat rat and monkey models of Parkinson’s disease^13^,^14^,^15^.

To further test this possibility, we looked at the characteristics of the pre-ictal theta band signal, which in Experiment 1 was very different between BMI-On and BMI-Off sessions, during the mixed sessions. In this latter case we found that, not only was the pre-ictal theta band signal potency now similar between BMI-On and BMI-Off seizure episodes (BMI-On: 30.50 ± 1.5 dB; BMI-Off:27.25 ± 2.2 dB; paired samples t test t = 1.452,df = 6; P = 0.1968, n.s.), but that the theta band amplitude signals obtained during the BMI-Off episodes were now closer to those measured during BMI-On episodes (also compare values in X axis in Fig. 2F to values in Fig. 3C). Lastly, analysis of low power theta band durations (which in experiment 1 were smaller for BMI Off episodes), also revealed that these were now similar between BMI-On and BMI-Off episodes (BMI-On: 2.31 ± 0.34 secs; BMI-Off: 2.44 ± 0.38 secs; Man-Whitney U = 163.5, P = 0.7084, n.s.). Therefore, these results suggest that, in this experiment, BMI-Off episodes where, to some extent, affected by DCS delivered during the BMI-On episodes.

### DCS is effective in both male and female rats

To identify possible gender specific differences in our results^23^, we further pooled male or female rats from both experiments and compared the main findings of this study according to animal gender. The use of DCS reduced the overall duration of seizure episodes in both male (t-test with Welch’s correction, t = 4.665, df = 59, P < 0.0001) and female rats (t-test with Welch’s correction, t = 3.563, df = 59, P = 0.0007). Additionally, the pre-ictal theta band signal was predictive of seizure episodes in both male (BMI-Off: F_1,42_ = 13.37; R^2^ = 0.24;P = 0.0007) and female rats (BMI-Off: F_1,33_ = 4.357; R^2^ = 0.12; P = 0.0447) when DCS was Off, but not when it was On (BMI-On Male: F_1,29_ = 0.42; R^2^ = 0.01;P = 0.52, n.s.; BMI-On Female: F_1,28_ = 0.30; R^2^ = 0.01;P = 0.59, n.s.).

## Discussion

We demonstrated here the efficacy of a closed-loop BMI that triggered DCS in response to pre-seizure and seizure patterns in LFP activity in PTZ-treated rats. Overall, we observed that our BMI quite effectively reduced the number of seizure episodes, and their duration, while also changing the overall pattern of LFP activity associated with the pre-ictal phase of PTZ-triggered seizures. Therefore, we propose that the main anti-seizure effect of DCS is obtained via the reduction in the pre-ictal theta band activity, a good predictor of seizure duration. Lastly, we found that our BMI was effective in both male and female rats, even though our experiments were not controlled for the estrous cycle^23^.

Previous studies have shown increases as well as decreases in epileptic related activity after treatment with DCS^19^,^24^. Here, we have specifically used DCS in response to a change in LFPs signal and consistently observed improvement in multiple physiological parameters. We attribute the differences between our findings and previous results to the fact that we delivered DCS only in response to LFP changes instead of stimulating indiscriminately^24^. Another important factor is that we only employed high frequency DCS in the present study, since we and others have observed (D.G.: personal observation) increased seizure activity when low frequency DCS was delivered^24^. Using transcranial electrical stimulation in a different model, Berenyi *et al.* have developed a closed loop BMI for epilepsy^21^. While that study was able to achieve reductions in seizure related activity somewhat higher than the ones achieved here, it is important to note that they used a different chemical agent. Future studies comparing different BMI approaches and epilepsy models will help identifying pros and cons, as well as efficacy, of each technique. At this point it is important to recall that the PTZ model – as used here - may not be the best animal model to represent the subset of patients with refractory epilepsy^20^. Therefore, the effects of our closed loop BMI will have to be further tested in other animal models of epilepsy.

It could be argued that the differences found between BMI-On and -Off seizure episodes reported here could be the result of differences originating from repeated PTZ administration. Although repeated administration of PTZ is often used as a model for chronic seizures (see Erkeç and Arihan 2015 for a review)^25^ our results cannot be explained by such effect alone. First, in experiment 1, not only BMI-On and -Off sessions were typically alternated, but some rats started with BMI-Off sessions while others started with BMI-On sessions. Second, there was no difference in seizure episodes (duration and frequency) in rats tested in the same conditions in consecutive sessions. Third, most PTZ kindling protocols involve more than 10 doses of PTZ^25^,^26^,^27^ or intervals of more than 20 days between the series of PTZ injections^28^. Lastly, results from experiment 2 (where the BMI was turned On and Off within the same session) further controlled for the possibility of differences in BMI-On and -Off sessions being the result of PTZ-induced kindling alone.

This study also reports, for the first time, that the pre-ictal theta band signal can be used as good predictor for seizure episode duration in PTZ-treated rats. It is not yet clear why this signal is related to the duration of the seizure episodes, but a possible explanation involves a mechanism where a state of seizure derives from an abnormal transition between brain states resulting from an imbalance between corticofugal inhibition and thalamocortical excitation. In healthy rodents, strong increases in theta band signal, with partial increases in other frequencies, are also present during whisker twitching^29^,^30^, a non-pathological seizure-like state controlled by S1 that is characterized by general immobility coupled with improved ability to detect incoming tactile stimuli. From this cortically controlled state, neural activity does not usually evolve to seizure episodes, but rather transitions to a state of quiet waking where the animal is either immobile or engaged in stereotyped behaviors^31^.

In a rat model of cortical injury generated epilepsy, an initial stimulus from the injured area to the thalamus will make the thalamocortical loop transition to a hypersynchronization state characterized by seizures^32^. This state is maintained by the thalamus and can be reversed by optogenetic thalamic stimulation^32^. Together, these findings suggest that many of the differences found in theta power in this and previous studies could be the result of this critical balance in the thalamocortical loop where S1 corticofugal inhibition^33^ maintains theta oscillations within a normal range (i.e. whisker twitching), but that otherwise, if theta oscillations become mostly dependent on a hyper excitable thalamus^32^,^34^, this state will then transition to a state of hypersynchronized seizure activity. Note that such a mechanism could additionally explain the differences found in previous DCS studies. Thus, if theta band activity critically reflects a balance between thalamic and cortical activity, electrical stimulation to the lemniscal pathway could result in thalamic increased excitability or, if sufficiently strong, it could in addition stimulate S1 and increase cortico-thalamic inhibition^33^. The differences in seizure related activity found when DCS was applied with low or high frequencies can be partially explained within this framework. Low frequency DCS would increase seizure activity^24^ because it should affect mostly thalamocortical synapses, while increasing stimulation frequency should be able to increase both the thalamus and S1 (Supplementary Figure S1), therefore activating the corticofugal synapses and improving seizure related activity (ref. 19 and here). Lastly, this critical balance between S1 inhibition and thalamic excitation could also explain the predictive power of the pre-ictal signal. The observation that S1 controls the state of whisker twitching (which is characterized by high potency theta oscillations) suggests that the bimodal distribution of the predictive pre-ictal theta signal found here may actually correspond to two different brain states resulting from the initial conditions imposed by the pre-ictal theta signal (one prone to long seizures and another one prone to short seizures). In this scenario, a theta signal smaller than −45 dB in S1 (Fig. 2F) after the injection of PTZ would constitute the critical potency required to disrupt the balance in the thalamocortical loop, transitioning to a state of long seizures, while a theta signal larger than −45 dB, while still disrupting the balance in the thalamocortical loop, would promote transition to a state where short seizures occur. It is important to note however that DCS activates multiple regions, making it unlikely that the proposed mechanism would be the only source for the brain state transitions described. Future studies involving recordings and stimulation across the thalamocortical loop will allow dissecting to what extent DCS affects this theta signal in each structure as well as its significance in different thalamocortical states.

Electrical neural stimulation has been used as an alternative to surgery for intractable epilepsy cases, primarily through deep brain stimulation, vagal nerve stimulation, and trigeminal nerve stimulation. Deep brain stimulation has presented an efficacy of up to 68% responders after 5 years^35^ but is extremely invasive and cannot be performed in many patients. Meanwhile, vagal and trigeminal nerve stimulation procedures, have demonstrated relatively low efficacy, with 49%^36^ and 50%^37^ respectively, and may have more side effects^38^ than deep brain stimulation. Thus, on one side, deep brain stimulation has achieved very good seizure reduction, but is extremely invasive and expensive, making it a solution for only a small fraction of the patients in need. On the other side, more peripheral stimulation procedures, which are much less expensive and invasive, have a much lower efficacy rate and are associated with increased side effects. Finally, DCS seems to rest in the middle ground between these other electrical neurostimulation alternatives, since it’s less invasive than deep brain stimulation and few side effects have been reported when DCS is used for other diseases^39^. Translating our findings into human patients will allow comparison of the efficacy rate between DCS and these other alternatives.

Previously we have shown that DCS ameliorates symptoms of Parkinson’s disease by desynchronizing pathological low frequency corticostriatal oscillations, therefore creating a brain state permissible for the initiation of locomotion in severely dopamine depleted rodents and non-human primates^13^,^15^. More specifically, high frequency DCS inhibited oscillatory neuronal activity synchronized at the beta frequency in the basal ganglia through activation of various structures along the dorsal column medial lemniscal pathway^15^. Our present PTZ results are in line with these previous Parkinson’s disease studies, by suggesting that DCS is responsible for the desynchronization of pathological synchronous activity characteristic of the PTZ model of epilepsy. In fact, this observation can be generalized to other disorders, since we and others have demonstrated that pathological synchronous activity seems to be the hallmark of pathological brain states recorded from multiple models of neurological and neuropsychiatric disorders such as Parkinson’s disease^13^,^14^,^15^, epilepsy (here and ref. 19), bipolar disorder^40^, and schizophrenia^41^. Therefore, based on this cumulative body of evidence showing abnormal timing in brain circuitry, we propose that the aforementioned diseases can all be classified within a broad spectrum of pathological timing brain states (i.e. hyper- or hyposynchronized), that resemble the basic neurophysiological hallmarks of epilepsy. While these diseases share excessive synchronization as a common feature, they differ in the type of neural circuits involved in each case^42^. A testable prediction of this hypothesis would be that any type of nerve stimulation capable of significantly altering the balance between regions responsible for these synchronizations, should also be able to induce at least a partial relief of symptoms in these disorders^42^,^43^.

In conclusion, we propose that DCS should be tested in other rodent and primate models of chronic epilepsy to measure its efficacy in controlling these pathological brain states over longer periods of time. These studies would be essential to determine the true potential of DCS as a non-pharmacologic alternative therapy for use in humans suffering from chronic, untreatable epilepsy.

## Methods

All animal procedures were performed in accordance with the National Research Council’s Guide for the Care and Use of Laboratory Animals and were approved by the Duke University Institutional Animal Care and Use Committee. Long Evans male and female rats weighing between 250–400 g were used in all experiments.

### Surgery for microelectrode array implantation

Animals went through two different surgeries: one to implant recording electrodes and the other to implant the stimulation electrodes. Recording electrodes: Fixed or movable microelectrode bundles or arrays of electrodes were implanted in the S1 of rats and additional regions (for the present study we did not evaluate the activity in other regions). Anesthesia was induced with 5% halothane, and maintained with ketamine (100 mg/kg), xylazine (10 mg/kg) and atropine (0.05 ml). Craniotomies for S1 recordings were made and arrays lowered at the following stereotaxic coordinates: [(AP) −3.5 mm, (ML), −5.5 mm (DV) −1.5 mm]^44^. Stimulation electrodes for spinal stimulation were also implanted under anesthesia as described above. Postoperative weight was monitored daily. The implantation procedure was performed as previously described^14^. Specifically, stimulation electrodes were inserted in the epidural space under thoracic vertebra T2 and, to prevent electrode migration, were tied to it with surgical suture.

### Electrophysiological recordings

A Multineuronal Acquisition Processor (64 channels, Plexon Inc, Dallas, TX) was used to record neuronal spikes, as previously described^45^. Briefly, neural signals were recorded differentially, amplified (20,000–32,000X), filtered (filtering band between 400 Hz and 5 kHz), and digitized at 40 kHz. Local field potentials (LFPs) were acquired by band-pass filtering the raw signal (0.3–400.0 Hz), preamplified (1,000), and digitized at 1,000 Hz using a digital acquisition card (National Instruments, Austin, TX) and a multineuronal acquisition processor (Plexon).

### Pentylenetetrazole administration

Each recording session, independently of the experiment, was performed on a different day. Both male and female rats were tested under the exact same conditions. PTZ (SIGMA Aldrich) administration was prepared by dilution of 100 mg/kg of PTZ in 1 ml saline. This was then administered IP under isofluorane anesthesia. As BMI sessions were preceded by an initial baseline recording period, rats injected with the PTZ could be immediately brought to the recording room with a delay of no more than 5 minutes. BMI sessions started approximately 5–10 minutes after the administration of PTZ. The recording sessions (in both experiments) lasted 60 minutes. In preliminary experiments we observed that PTZ effects were less variable within the first 60–90 minutes.

### Data analysis

Neuronal data obtained from a total of 30 recording sessions was processed and analyzed using NeuroExplorer (version 3.266; NEX Technologies, Madison, AL) and custom scripts written in Matlab (12.0; Mathworks, Natick, MA). A seizure episode was defined as a period where observable muscle spasms and high amplitude oscillations in raw LFP trace, were accompanied by increased power across multiple frequency bands. Seizure episodes were initially identified during the session using both behavior and raw LFP traces as indicators, and later confirmed through detailed reanalysis of raw LFP traces and spectrograms. Comparison of seizure and SWD frequencies (calculated in seizure episodes or SWD events per minute) was made using a paired samples t test or Wilcoxon signed ranks test. When an animal had more than one BMI-On or BMI-Off session, a single value resulting from the mean of the sessions was used for comparison. Seizure duration was compared using the Mann-Whitney test. Analysis of the overall distribution of seizure durations indicated a bimodal distribution. Accordingly, seizure episodes were analyzed as Long (≥60 secs) or Regular (<60 secs). Then the proportion of Regular and Long seizure episodes was calculated for BMI-On and BMI-Off episodes. Lastly, the proportion of Regular and Long seizure episodes was compared using Fisher’s exact test. These calculations were performed separately for each experiment. For comparison of pre-ictal theta band spectrogram power we used values from 4.5–8 Hz frequencies in the 5 seconds before the timestamp that was identified as the start of the seizure episode. Theta power was calculated from the original signal processed in Neuroexplorer, followed by processing with custom scripts written in Matlab. Values were normalized with the Log of power spectral density (dB) and initially analyzed in bins of 100 ms. Calculation of theta power for correlation was made using a single 5 second bin (the 5 seconds immediately before seizure onset) for theta frequency that was then correlated to seizure episode duration. For ease of presentation, spectrograms are presented in bins of 100 ms and smoothed with a Gaussian filter of 300 ms. Statistical comparison of pre-ictal theta band power was made using an independent (Experiment 1) or paired (Experiment 2) samples t test. Pearson correlation was calculated using seizure episode duration and the pre-ictal theta power. The duration of theta band potency decrease was compared using data from the spectrogram of the whole session initially processed in 1 second bins in Neuroexplorer. A Zscore was calculated for theta band frequency for each bin across the session, and then periods of 5 seconds before the onset of each seizure episode were analyzed. As increased theta band Zscores were present almost exclusively during seizure episodes or spike-and-wave discharges, we analyzed instead periods where Zscores decreased (i.e. indicating a low potency theta band signal). Decreases in theta potency were considered here as a Zscore equal or below 1.0 standard deviation. The duration of each response was then considered as the number of consecutive bins where the potency of the signal corresponded to this criterion. Lastly, duration of low theta power responses was compared between BMI-On and BMI-Off episodes in each separate experiment, using the Mann-Whitney U test.

### Brain-machine interface based on Dorsal Column Stimulation

Our brain-machine interface used Dorsal Column Stimulation (DCS) cues that were generated by an electrical microstimulator (Master 8. AMPI, Jerusalem, Israel) controlled by a custom Matlab script (Natick, USA) receiving information from a Plexon system over the internet. This real-time neural analysis and stimulation system has been previously described for a different purpose^46^,^47^. Here, we have pre-determined for each rat, a threshold in raw LFP traces that was typically crossed only in the presence of LFP epileptic activity (i.e. spike and wave discharges or seizure episodes). Upon detection of such threshold crossing, a pattern of 200 (bipolar, biphasic, charge balanced; 200 μsec) pulses at 500 Hz was delivered to the dorsal column of the spinal cord at the level of T1-T2 segments. Current intensity varied from 100–200 μA. Seizure episodes where DCS failed to stimulate for at least 75% of the episode duration were considered as ‘Partial’ stimulation and were excluded from final analysis. These included a total of 6/53 = 11.32% episodes in Experiments 1 and 2.

In Experiment 1 rats were typically tested in BMI-On and BMI-Off sessions on alternate days. Similarly, in Experiment 2, rats seizure episodes with BMI-On were alternated with BMI-Off episodes. Changes to these pre-established conditions were made when technical problems occurred (e.g. incomplete session, cable disconnecting, noise, inadequate threshold etc.).

## Additional Information

**How to cite this article**: Pais-Vieira, M. *et al.* A Closed Loop Brain-Machine Interface For Epilepsy Control Using Dorsal Column Electrical Stimulation. *Sci. Rep.* **6**, 32814; doi: 10.1038/srep32814 (2016).

## Supplementary Material

Supplementary Information
