# Supplementary material for: A Closed Loop Brain-machine Interface for Epilepsy Control Using Dorsal Column Electrical Stimulation
Source: Sci Rep. 2016 Sep 8;6:32814. doi: 10.1038/srep32814 (PMC5015048; doi:10.1038/srep32814)
Supplement: Supplementary Information [file srep32814-s1.pdf]

Supplementary Information

A CLOSED LOOP BRAIN-MACHINE INTERFACE FOR EPILEPSY CONTROL USING DORSAL COLUMN ELECTRICAL STIMULATION

Miguel Pais-Vieira[1,5,6]*, Amol P. Yadav[1,2]*, Derek Moreira[1], David Guggenmos[1], Amílcar Santos[1],Mikhail Lebedev[1,4], Miguel A.L. Nicolelis[1,2,3,4, 7]

Department of Neurobiology[1], Department of Biomedical Engineering[2], Department of Psychology and Neuroscience[3], Duke Center for Neuroengineering[4], Duke University, Durham, NC 27710; Centro de Investigação Interdisciplinar em Saúde, Instituto de Ciências da Saúde, Universidade Católica Portuguesa, Porto, Portugal[5], Instituto de Ciências da Vida e da Saúde, Universidade do Minho, Braga, Portugal[6]; Edmond and Lily Safra International Institute of Neuroscience of Natal[7], Natal, Brazil.

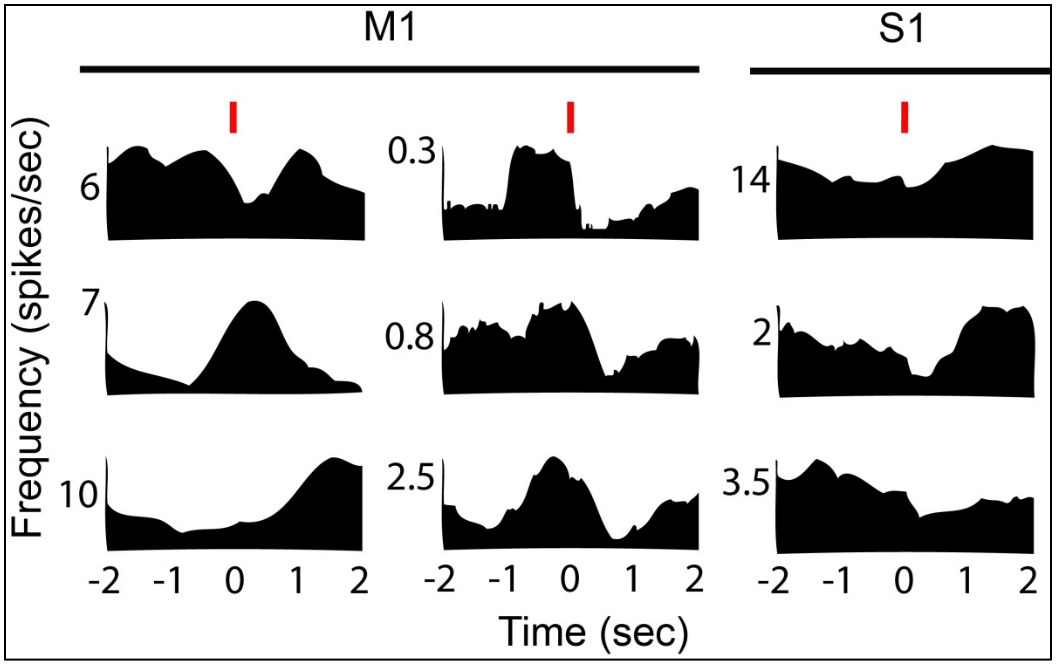

Supplementary Figure 1 – DCS modulates cortical structures. Examples of perievent histograms from neurons recorded from primary motor cortex (left and center columns) and from primary somatosensory cortex (right column). DCS – delivered at time t=0 secs induced increases or decreases in activity. In some neurons pre-stimulus modulation can be observed because DCS was delivered in trains of pulses.
